# Comparative osteoconductivity of bone void fillers with antibiotics in a critical size bone defect model

**DOI:** 10.1007/s10856-020-06418-1

**Published:** 2020-08-25

**Authors:** Rema A. Oliver, Vedran Lovric, Chris Christou, William R. Walsh

**Affiliations:** Surgical and Orthopaedic Research Laboratories, UNSW Sydney, Prince of Wales Clinical School, Prince of Wales Hospital, Level 1 Clinical Sciences Building, Randwick, NSW Australia

## Abstract

The study aimed to evaluate the comparative osteoconductivity of three commercially available bone void fillers containing gentamicin with respect to new bone, growth, host tissue response and resorption of the implant material. Defects were created in the cancellous bone of the distal femur and proximal tibia of 12-skeletally mature sheep and filled with three commercially available bone void fillers containing gentamicin (Stimulan-G, Cerament-G, Herafill-G). Peripheral blood was taken pre-operatively and at the time of implantation, as well as at intermittent timepoints following surgery to determine systemic gentamicin levels (5-,15- and 30- minutes, 1, 2, 3, 6, 12, 24, 48- and 72-hours, 3-, 6- and 12-weeks). Decalcified, embedded samples were stained with haematoxylin and eosin (H&E) and used to assess the host tissue response and the formation of new bone in the presence of test implant materials. No adverse reactions were noted at harvest at any time points for any cancellous implantation sites with the various implant materials. Comparative microCT analysis of the Stimulan-G, Cerament-G and Herafill-G test materials revealed a similar increase in bone surface area and volume between animals implanted with Stimulan-G or Cerament-G test materials. Animals implanted with Herafill-G test materials demonstrated the lowest increases in bone volume and surface area of the test materials tested, at levels similar to the negative control sites. By 12-weeks, Stimulan-G defects were completely closed with mature bone and bone marrow whilst the Cerament-G material was still present after 12 weeks by histological examination. In conclusion, this study demonstrated differences in the bone regenerative capacity of a range of bone void fillers in an in vivo setting.

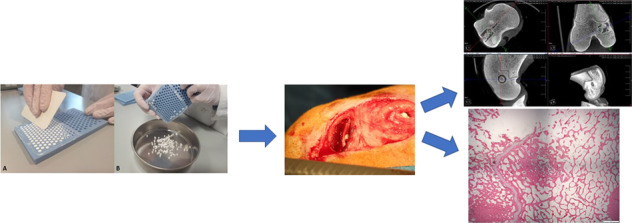

## Introduction

It is important to consider how to manage dead space following the management of infection in cases of periprosthetic joint infection or osteomyelitis. Following infection eradication, osseous repair of the cavity is critical following debridement. Patients with residual dead space are at high risk for subsequent infection or reinfection [1] therefore in the setting of active infection, methods which support the local release of antibiotics are preferable.

Poly-methyl methacrylate (PMMA) is a cement that has been successfully used as a void filler in combination with antibiotics for over 40 years [2]. PMMA mixed with antibiotic powder serves as a local antibiotic delivery vehicle. PMMA is a non-absorbing space filler, which can load bear but requires surgical removal. For indications where structural integrity is less important than the ability to release antibiotics locally, the development of absorbable alternatives has gained momentum in recent years. Applications such as bone void fillers, as part of a dead space management programme are amongst those reporting promising outcomes [3–6].

There are many biodegradable bone void fillers, largely based on either calcium sulfate or calcium phosphate. Calcium sulfate bone graft substitutes play a major role in the surgical management of dead space thanks to their biocompatibility, porosity and biodegradability [7]. The osteoconductive properties of calcium sulfate have been established since 1892 [8]. Previous work has shown that the material provides an osteoconductive scaffold for a number of cell types including blood vessels and osteogenic cells [9, 10] and can stimulate new bone formation at a rate comparable with autogenous bone [11–14]. Moreover, it is also a suitable carrier for aminoglycoside antibiotics [14–16]. As such, it is often used in the management of bone infection, leading to the development of numerous commercially available derivatives.

The main constituent of bone is calcium phosphate. Calcium phosphate includes a family of minerals containing calcium ions together with orthophosphates [17]. The material can exist in a number of forms such as tricalcium phosphate (TCP) or hydroxyapatite [17], which is recognised as the most abundant form of calcium phosphate, making around 70% of the mineral content of bone by weight [18].

A number of commercially available bone void fillers exist which combine more than one type of calcium salt (phase) such as the biphasic geneX® (Biocomposites Ltd, UK) containing a combination of β-tricalcium phosphate and calcium sulfate or CERAMENT (BONE SUPPORT AB, Ideon Science Park, Sweden) a biphasic composite of calcium sulfate and hydroxyapatite. In addition, a number of commercially available triphasic compounds also exist combining calcium sulfate with Brushite and β-tricalcium phosphate such as with PRO-DENSE™ (Wright Medical, US). Other biodegradable materials which are clinically used in bone infection include allograft bones [19], collagen implants [20] and bioactive glass [21].

An ideal bone graft substitute has certain characteristics of biocompatibility, bioresorbability, osteoconductivity and osteoinductivity which are important for bone proliferation and bone healing, by providing an environment suitable for osteoblast proliferation [22]. Moreover, it is important for any material to carry out its function in the absence of any adverse local or systemic effects [23] (Table 1). A number of commercially available bone cement and bone void fillers exist which claim to have osteoconductive properties. The aims of this study were to evaluate the comparative osteoconductivity of three of these commercially available bone void fillers containing gentamicin with respect to new bone, growth, host tissue response and resorption of the implant material. In addition, the systemic gentamicin levels following implantation of materials was also evaluated.

**Table 1:** Ideal properties of a bone void filler

Ideal properties of a bone void filler if infection is present or suspected
Biocompatible to avoid local reactions
Bioabsorbable to avoid the need for removal surgery
Able to elute high levels of antibiotic
Able to provide mechanical strength to support bone
Osteoconductive to encourage growth new bone overgrowth and remodelling

Adapted from [23]

## Materials and methods

### Preparation of implant materials

Three commercially available bone graft materials were assessed in this study as detailed in Table 2. Calcium sulfate alpha-hemihydrate powder (Stimulan® Rapid Cure, Biocomposites Ltd. UK) was used to prepare beads for implantation. Twenty grams of calcium sulfate hemihydrate were mixed with 240 mg Gentamicin Sulfate (supplied as a liquid at a concentration of 80 mg/2 mL) (Amdi-Pharm) (Stimulan-G). CERAMENT™ | G (CERAMENT™ | GENTAMICIN gentamicin paste+ CERAMENT™ | MIXING LIQUID 9 mg/ml saline, BONE SUPPORT AB, Ideon Science Park, Sweden) was prepared according to the Instructions for Use (IFU). For both Stimulan-G and Cerament-G, all materials were mixed for 30 to 60 sec to form a smooth paste, and then set into 3.0 mm diameter hemispherical beads in a flexible mold. Herafill® beads G (Heraeus Medical GmbH, Wehrheim, Germany) (Herafill-G) were supplied as (6 × 6 mm) calcium sulfate/calcium carbonate composite beads containing 1% gentamicin sulfate (2.5 mg gentamicin base) as an antibacterial agent. A control material (geneX®, Biocomposites Ltd, UK) containing no antibiotics was prepared in accordance with the manufacturer’s instructions [24] (Table 2).

**Table 2:** Materials tested

Material composition	Commercial name	Manufacturer
100% Calcium Sulfate	Stimulan Rapid Cure	Biocomposites Ltd, UK
60% Calcium Sulfate 40% Hydroxyapatite	Cerament-G	Bone Support AB, Sweden
72% Calcium Sulfate 18% Calcium Carbonate 9% Hydrogenated Triglyceride	Herafill-G	Heraeus Medical GmbH
50% Calcium Sulfate 50% β-tricalcium phosphate	geneX (Control)	Biocomposites Ltd, UK

### Surgery

The implant materials were evaluated using 12-skeletally mature sheep (2.5 years old ± 0.5, body weight 60–75 kg). Ethical approval was obtained through the Animal Care and Ethics Committee of the University of South Wales (ACEC#: 15/82 A). Animals were acclimatised for at least seven days prior to surgical procedures. Following gaseous anaesthesia, 13 × 10 mm defects were created into the cancellous bone of the distal femur and proximal tibia. In each animal, four defects were created (right and left-hand side). For the test animals, beads of implant materials were implanted directly into each defect and the periosteum reflected and the skin closed. Each animal received the same material and antibiotic concentration to examine the in vivo release kinetics of antibiotics from implant materials in the serum versus time. Defects in the control animals were assigned positive and negative controls. In the case of the negative controls, the defect remained unfilled. For the positive controls, defects were filled with an absorbable calcium sulfate/calcium phosphate composite bone graft (geneX®, Biocomposites Ltd, UK). In summary, three time points (3, 6 and 12 weeks) were utilized, with one sheep per material per time point, each with 4 defects (except for the empty and positive control groups which had two defects per sheep). A detailed description of the test materials and implant sites is outlined in Table 3. Animals were given post-operative analgesic relief (4mls of Carprofen, SC) for the first two days following surgery and thereafter, as required, following clinical examination. At each time point, four sheep were sacrificed according to the study design, tissues harvested for analysis and explanted bones evaluated by means of radiography and microCT to characterize the extent of bone regeneration. This was performed prior to histological processing due to its non-destructive nature. The general integrity of the skin was noted along with the reaction to the underlying subcutaneous tissues and identified as normal or abnormal.

**Table 3:** Implantation schedule

Cancellous implantation site					
Animal	Time	LF	LT	RF	RT
W2735	12 weeks	Stimulan-G	Stimulan-G	Stimulan-G	Stimulan-G
W2736	12 weeks	Cerament-G	Cerament-G	Cerament-G	Cerament-G
W2737	12 weeks	Herafill-G	Herafill-G	Herafill-G	Herafill-G
W2738	12 weeks	geneX	Control	Control	geneX
W2739	6 weeks	Stimulan-G	Stimulan-G	Stimulan-G	Stimulan-G
W2740	6 weeks	Cerament-G	Cerament-G	Cerament-G	Cerament-G
W2741	6 weeks	Herafill-G	Herafill-G	Herafill-G	Herafill-G
W2742	6 weeks	geneX	Control	Control	geneX
W2743	3 weeks	Stimulan-G	Stimulan-G	Stimulan-G	Stimulan-G
W2744	3 weeks	Cerament-G	Cerament-G	Cerament-G	Cerament-G
W2745	3 weeks	Herafill-G	Herafill-G	Herafill-G	Herafill-G
W2746	3 weeks	geneX	Control	Control	geneX

Table above lists the implantation schedule for all twelve animals used in this study. In the case of Stimulan-G, Cerament-G and Herafill-G test groups, all four defects in one animal were all implanted with the same material. For the control animals, two defects were allocated as empty controls (negative control) while two defects were filled with geneX paste (control)

*LF* Left Femur, *LT* Left Tibia, *RF* Right Femur, *RT* Right Tibia

### Radiography

Harvested bones were X-rayed using a Faxitron (Faxitron, Wheeling, IL) and digital plates (AGFA, Sydney Australia). Bones were X-rayed in the anteroposterior and lateral views to determine the in vivo resorption of implant materials.

### MicroCT

MicroCT was performed using an in vivo scanner (Inveon, Siemens Medical, PA, USA) at a resolution of 53.12 µm. Three-dimensional models of the bones were created using image analysis software (Inveon Research Workplace [IRW], Siemens Medical Solutions, Knoxville, Tennessee, USA). Images were examined in the axial, sagittal and coronal planes for the presence of residual implant material or evidence of new bone formation. The 3D indices analysed in the defined region of interest (ROI) were bone volume (BV) and bone surface area (SA).

### Decalcified histology

Samples for histology were fixed in neutral buffered formalin (10%) for 48 h and decalcified in 10% formic acid solution. The decalcified, embedded samples were sectioned mediolaterally to 5 microns using a microtome (Lecia, Germany). Slides were stained with haematoxylin and eosin (H&E). The stained sections were examined under light microscopy (Olympus, Japan). Images of the histology were digitally captured (Olympus DP72 Camera) and used to assess the host tissue response and the formation of new bone in the presence of test implant materials at the implant site/ host tissue boundary.

### Peripheral blood analyses

**Fig. 1:**
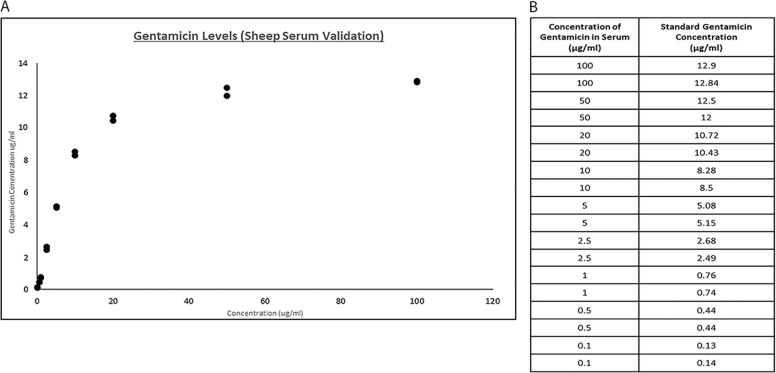
**a**, **b** Sheep serum validation with known concentrations of gentamicin. Dose response curve (**a**) and data (**b**) obtained for known gentamicin levels in sheep serum. Gentamicin concentrations of 0.1, 0.5, 1, 2.5, 5, 10, 20, 50 and 100 µg/ml were prepared to provide a standard curve

Gentamicin concentrations of 0.1, 0.5, 1.0, 2.5, 5.0, 10, 20, 50 and 100 µg/ml were used to generate a standard curve of gentamicin in sheep serum (Fig. 1). This standard curve was used to determine the limits of detection (LoD) for the test. A standard curve of gentamicin in saline was also run concurrently for comparison. This portion of the study was carried out with a standardised assay. Quantification of gentamicin concentration was carried out using a Roche Cobas 6000 analyser (Roche Diagnostics, Mannheim, Germany) (Gentamicin Assay Range: 0.4–10.0 µg/ml, Functional Sensitivity 0.4 µg/ml). Peripheral blood was taken pre-operatively and at the time of implantation (0 min) as well as 5-, 15- and 30-minutes following surgery, 1, 2, 3, 6, 12, 24, 48- and 72-hours following surgery and 3, 6 and 12 weeks throughout the study for standard blood panel hematology/biochemistry (IDEXX Laboratories, Sydney, Australia) and to determine systemic gentamicin levels.

## Results

### Surgery

**Fig. 2:**
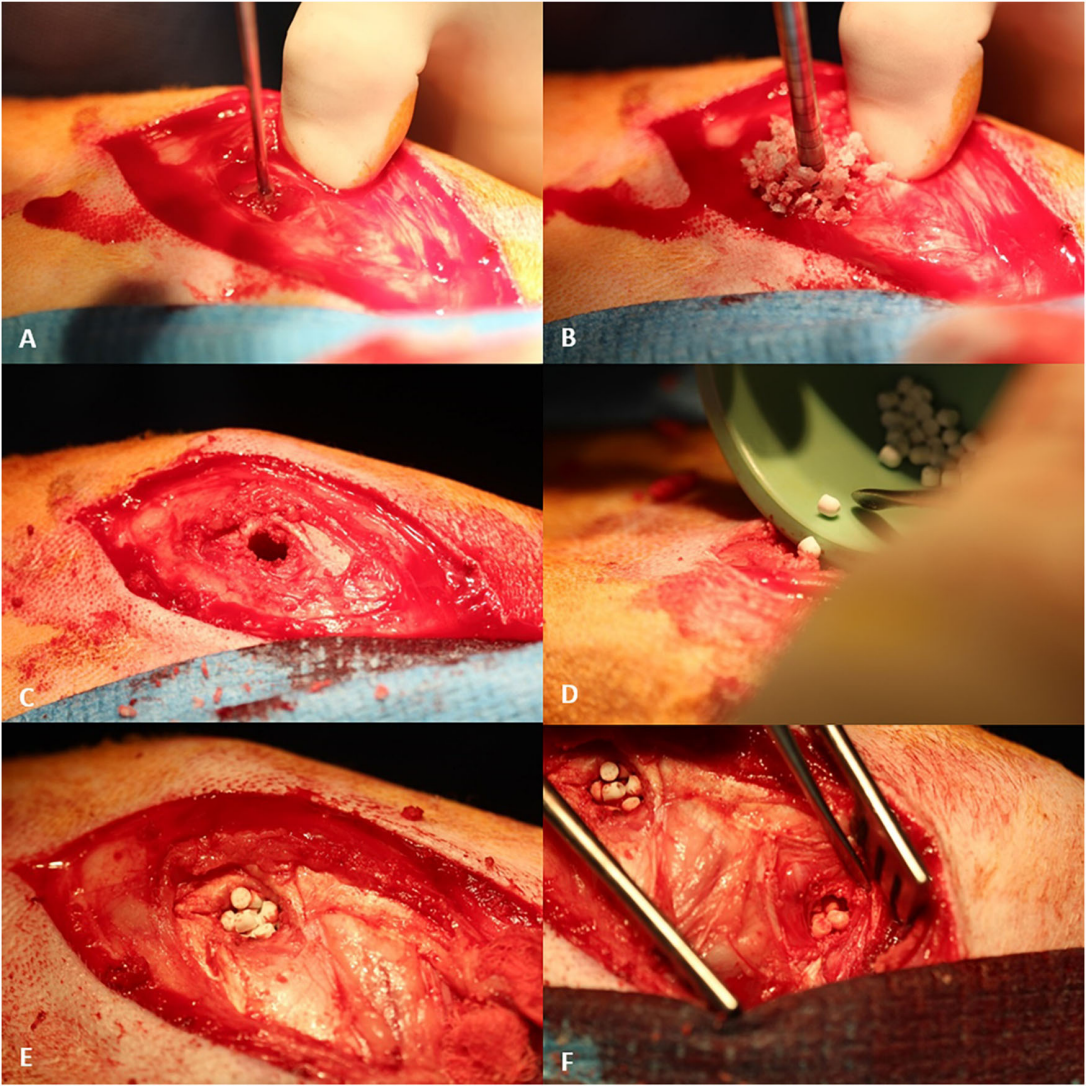
**a**–**f** Representation of the surgical procedure and the implantation of the test materials. Cylindrical defects of 11 mm in diameter and 20 mm deep were placed with a cannulated surgical acorn drill bit bilaterally in the cancellous bone within the epiphyseal region of the proximal tibia and distal femur in each sheep. The test/control materials were implanted, and the wounds closed in a routine fashion

Surgery was completed without any adverse events. The sheep were monitored daily during the first week and weekly thereafter specifically looking for signs of swelling, infection, bleeding, haematoma or general ill health. All animals recovered well after surgery. A representation of the surgical procedure and implantation of the test materials can be seen (Fig. 2a–f).

### Blood serum gentamicin levels

Systemic levels of gentamicin were detected in the serum of sheep implanted with Stimulan-G beads, 1-hour post-implantation, peaking at 3 h post-implantation with an average concentration of 1.07 µg/ml (Table 4). Serum levels of gentamicin then dropped in subsequent time points, with detectable serum levels in two animals, 72 h post-implantation (average 0.30 µg/ml) and no detectable gentamicin in the serum of any animals implanted with Stimulan-G in all subsequent time points (Table 4). A similar trend was detected in sheep implanted with Cerament-G beads. Systemic levels of gentamicin were detected in the serum of a sheep implanted with Cerament-G beads, 30 mins post-implantation, with levels of gentamicin peaking at 3 h post-implantation with an average concentration of 0.72 µg/ml (Table 4). Serum levels of gentamicin then dropped in subsequent time points to 72 h post-implantation (average 0.29 µg/ml). No gentamicin was detected in the serum of sheep implanted with Cerament-G beads at 3, 6- or 12-weeks post-implantation. For sheep who were implanted with the Herafill-G beads, systemic levels of gentamicin were detected in the serum immediately after implantation (Table 4). Systemic levels of gentamicin continue to increase in subsequent time points, to 24 h, at which point systemic levels of gentamicin peaked in the serum, with an average concentration of 0.46 µg/ml. Serum levels of gentamicin then dropped in subsequent time points. Interestingly, systemic levels of gentamicin were still detected in the serum of sheep implanted with Herafill-G beads, 12 weeks post implantation (0.49 µg/ml) (Table 4). No antibiotic levels were detected in the control animals.

**Table 4:** Summary of gentamicin levels

	Test material		
	Stimulan-G	Cerament-G	Herafill-G
Pre-Op			
Implant			0.14 µg/ml
5 mins			0.16 µg/ml
15 mins			
30 mins		0.13 µg/ml	
1 h	0.29 µg/ml		0.16 µg/ml
2 h	0.84 µg/ml	0.62 µg/ml	0.15 µg/ml
3 h	1.07 µg/ml	0.72 µg/ml	0.16 µg/ml
6 h	0.75 µg/ml	0.70 µg/ml	0.34 µg/ml
12 h	0.18 µg/ml	0.36 µg/ml	0.37 µg/ml
24 h		0.15 µg/ml	0.46 µg/ml
48 h	0.30 µg/ml	0.47 µg/ml	0.35 µg/ml
72 h	0.30 µg/ml	0.29 µg/ml	
3 weeks			0.14 µg/ml
6 weeks			
12 weeks			0.49 µg/ml

Table above represents a summary of gentamicin levels for each test material group at each of the blood sampling intervals (µg/ml). Data was averaged for all the animals within each blood sampling interval. Empty spaces indicate no detection of gentamicin across any specimens treated within a specific test material group

### Limb harvesting

The animals were euthanized and the right and left hind limbs were harvested and photographed using a digital camera at their allocated time points. The surgical sites were examined for signs of adverse reaction or infection. The right and left femurs and tibias were isolated free of soft tissue and the surgical sites were also photographed using a digital camera. No adverse reactions were noted at harvest at any time points for any cancellous implantation sites with the various implant materials. All skin tissue and subcutaneous tissue appeared normal.

### Radiography

**Fig. 3:**
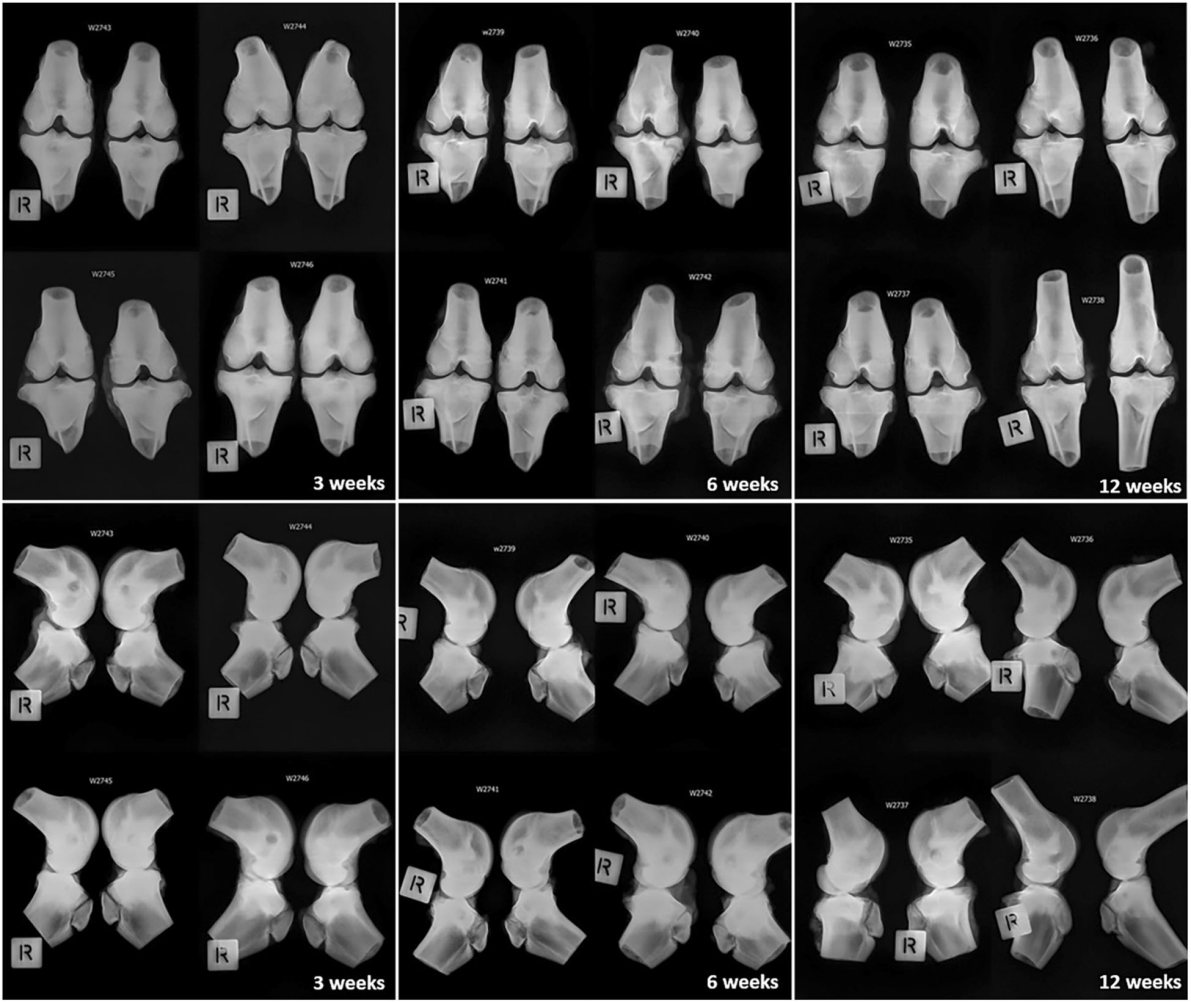
Anteroposterior (Top) and Lateral (Bottom) X-Rays. Representative X-Ray images of the right and left hind limbs throughout the duration of the study. Anteroposterior (AP) and Lateral (LAT) images were taken at 3, 6- and 12-weeks post-implantation to record the in vivo absorption of implant materials

Anteroposterior (AP) and Lateral (LAT) radiographs were used to visualise the appearance of implant materials in the bone. No adverse reactions were noted in the radiographs. While a progression in resorption of the materials could be noted by 12 weeks, no significant differences between the groups could be made due to the lack of detail with this endpoint as would be expected. Representative radiograph images can be seen in Fig. 3.

### MicroCT

**Fig. 4:**
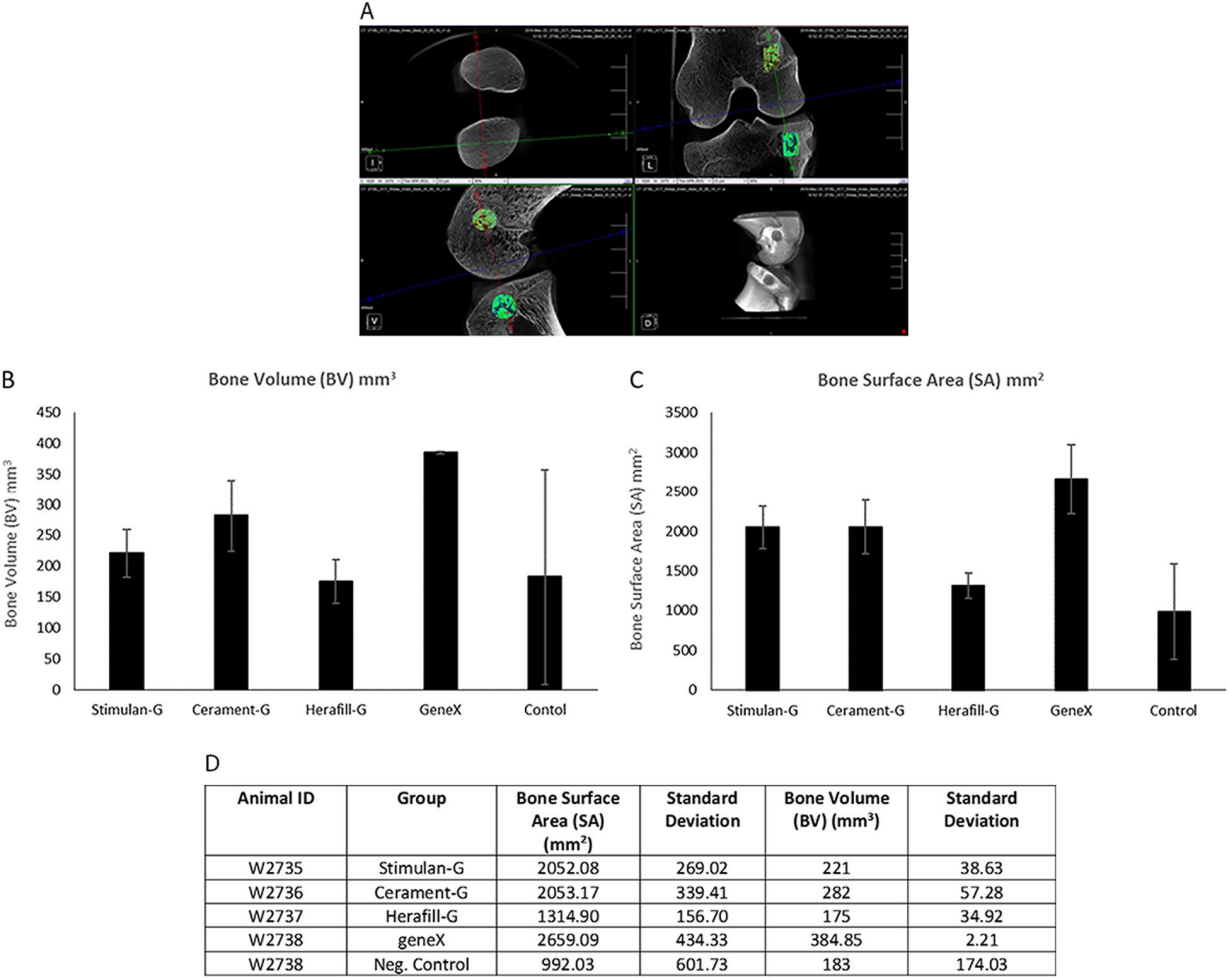
**a**–**d** Mean Bone Volume (BV) and Surface Area (SA) for each group (mean ± standard deviation). One hundred and twenty-five consecutive slices, in the centre of the defect were selected and assigned to a Region of Interest (ROI) shown in green. Representative image can be seen in **a**. Once the ROI was defined, the threshold values were adjusted to isolate the newly formed bone from bone marrow. The ROI was used to determine the bone volume (BV) and surface area (SA) of new bone. Bone Volume (**b**) and Surface Areas (**c**) were quantified using an IRW analysis tool by selecting a region for each implant under a constant threshold value. geneX and control samples (*n* = 2). Descriptive statistics (**d**) showing results (mean and standard deviations) of µCT analysis for Bone Surface Area and Bone Volume for each defect at the 12-week time point. Error bars show standard deviation

Micro Computed Tomography (microCT) provided an assessment of implant resorption and bone formation in the axial, sagittal and coronal anatomical planes and were used to confirm radiographic findings. Following 3D visualization, models were created using a threshold analysis technique to calculate the volume and the surface area of the newly formed bone. One hundred and twenty-five consecutive slices, in the center of the defect were selected and assigned to a Region of Interest (ROI). A representative image can be seen in Fig. 4a. This technique standardised the volume chosen for analysis across all defects. ROIs were created separately for each defect. Once the ROI was defined, the threshold values were adjusted to isolate the newly formed bone from bone marrow. Due to the presence of the residual material at weeks 3 and 6, this analysis was only performed at the 12-week time point. Bone Volume (BV) and Surface Areas (SA) were quantified using an IRW analysis tool by selecting a region for each implant under a constant threshold value (Fig. 4b–d). Three-dimensional analysis indicated that the sites implanted with the control geneX material demonstrated the greatest increase in both bone volume and surface area (384.55 mm^3^ and 2659.09 mm^2^ respectively). Comparative analysis of the Stimulan-G, Cerament-G and Herafill-G test materials revealed a similar increase in bone surface area and volume between animals implanted with Stimulan-G or Cerament-G test materials (2052.08/2053.17 mm^2^ and 221.24/282.78 mm^3^ respectively) (Fig. 4b–d). Animals implanted with Herafill-G test materials demonstrated the lowest increases in bone volume and surface area of the test materials tested, at levels similar to the negative control sites in which the defect remained unfilled (Fig. 4b–d).

### Histology

#### 3 weeks

**Fig. 5:**
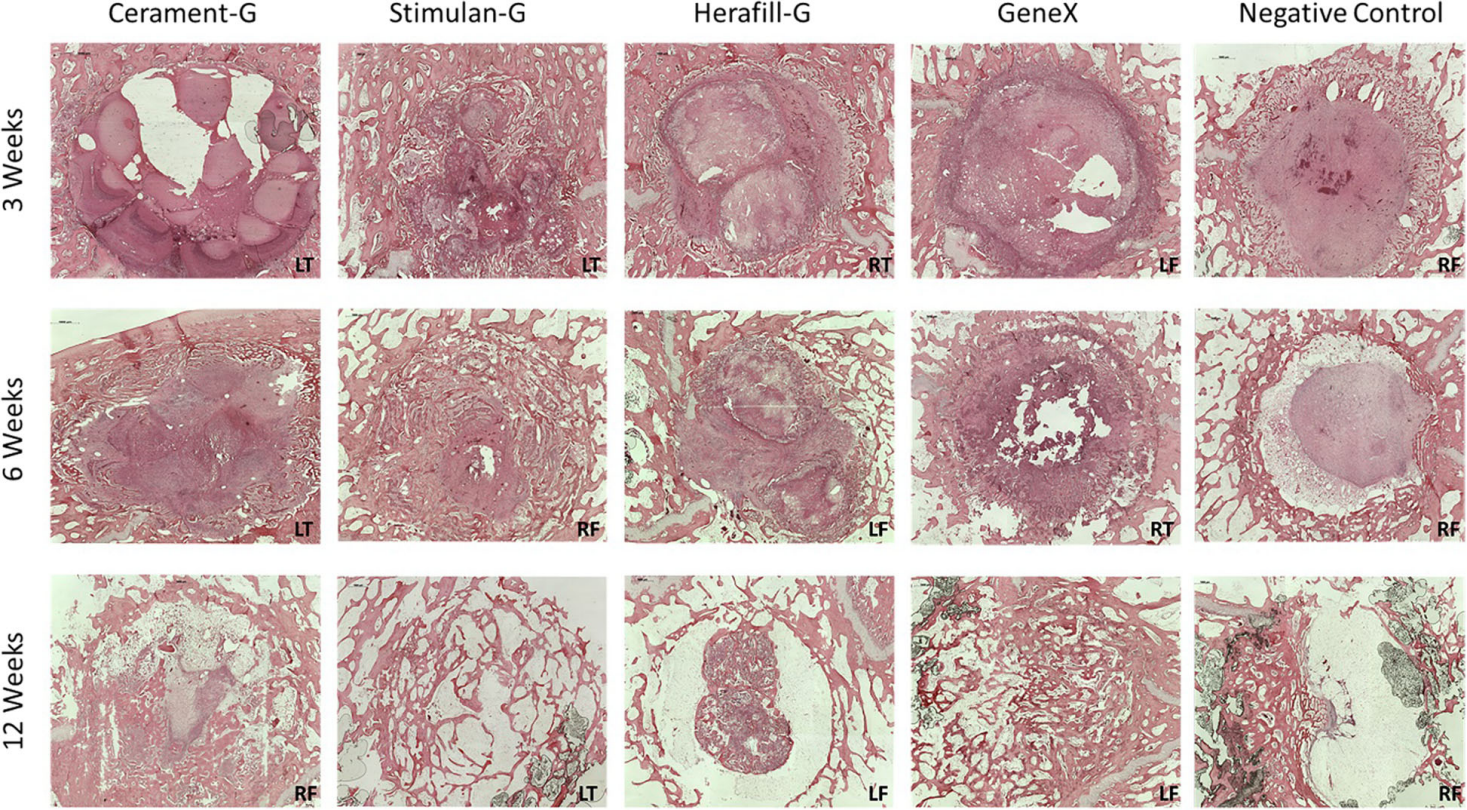
Histology of Bone after exposure to implant materials. Representative images of the histology from the right femur (RF) or left femur (LF) of sheep implanted with each of the test materials (Stimulan-G, Cerament-G, Herafill-G) or controls (geneX/ empty) at 3, 6 and 12 weeks. Slides were stained with H&E for microscopic analysis for bone reaction

Within three weeks, Stimulan-G filled defects had large amounts of newly formed bone at the margins of the defect. Residual material was noted centrally in the defect along with large amounts of fibrous tissue. In contrast, small amounts of newly formed bone were noted in the margins of defects filled with Cerament-G beads. Dense fibrous tissue was dispersed throughout the defect (Fig. 5). The Herafill-G filled defects were filled with residual material that had begun to absorb. The centre of the residual material remained acellular, with some new woven bone noted at the margins. Defects filled with the osteoconductive geneX (positive control) were filled at 3 weeks, with some new woven bone at the margins. In contrast, the control (empty) defects were empty at 3 weeks with some evidence of new bone woven at the margins, whilst the defect was filled with fibrous tissue (Fig. 5).

#### 6 weeks

After 6 weeks, the Stimulan-G filled defects were filled with newly formed bone. Small amounts of residual material were still present which was mainly covered in bone (Fig. 5). Some fibrous tissue was seen throughout the defect. By comparison, defects filled with Cerament-G were not closed and large amounts of the material was still present after 6 weeks. New bone formation continued from the margins of the defect towards the centre. The Herafill-G defects were also filled with residual material that had continued to absorb between 3 and 6 weeks. The centre of the residual material remained acellular. Some new woven bone was noted at the margins. New bone formation progressed between 3 and 6 weeks for defects filled with geneX (positive control). Large portions of the defect were filled with newly laid woven bone (Fig. 5). The material had continued to absorb and was isolated to the centre of the defect, along with dense fibrous tissue. In contrast, the control (empty) defects remained empty at 6 weeks, with evidence of new woven bone concentrated at the margins. Many of the defects remained filled with dense fibrous tissue (Fig. 5).

#### 12 weeks

By the 12-week time period, Stimulan-G defects were completely closed with mature bone and bone marrow. Only very small amounts of residual material remained (Fig. 5). By contrast, whilst the Cerament-G material continued to absorb between 6 and 12 weeks, there was still residual material present after 12 weeks. Bone marrow and mature bone were noted; however, the defects were not completely filled. Residual material was also present for defects filled with Herafill-G material, albeit at the centre of the defect which remained acellular (Fig. 5). Defects filled with geneX positive control were mostly filled with new bone at 12 weeks. Very small amounts of residual material were present (similar to Stimulan-G) which was concentrated within the newly formed bone. Bone marrow was present throughout the entire defect indicating maturation of the newly formed bone. By contrast, the control (empty) defects remained largely empty, with some bone formation found at the margins and bone marrow dispersed throughout the defect (Fig. 5).

## Discussion

This study looked to evaluate the comparative osteoconductivity of three commercially available bone void fillers containing gentamicin with respect to new bone, growth, host tissue response and resorption of the implant in a critical-size defect, non-infectious bone healing model. Inflammatory reactions have previously been described with the use of calcium sulfate-based bone void fillers [8, 25, 26]. No adverse reactions were noted at harvest at any time points for any cancellous implantation sites with the various implant materials. All skin tissue and subcutaneous tissue appeared normal. Anteroposterior (AP) and Lateral (LAT) radiographs were used to visualise the appearance of implant materials in the bone as per schedule at the time of harvest. No adverse reactions were noted in the radiographs.

The model evaluated a number of bone void fillers in combination with gentamicin. Previous reports of systemic gentamicin toxicity from local antibiotic carriers have been documented in infected hip arthroplasty [27] and in another study using demineralised bone matrix containing gentamicin [28]. In a contrasting study investigating the release profile of gentamicin from a calcium sulfate carrier in 20 patients with osteomyelitis, serum antibiotic levels remained low [29]. Systemic levels of gentamicin were detected in the serum of sheep implanted with Stimulan-G beads or Cerament-G beads, 1-hour post-implantation, peaking at 3 h post-implantation and dropping to no detectable levels 72-hours post implantation. These findings are in line with previous work in similar rabbit and canine models, which showed that systemic antibiotic levels were low and only detectable the first day after implantation and remained undetectable thereafter [30–32]. Conversely, systemic levels of gentamicin were still detected in the serum of sheep, 12 weeks post implantation with Herafill-G beads. This is in line with a previous study by Pforringer and colleagues who showed that systemic levels of gentamicin remained high following incorporation of Herafill-G beads in a rabbit model [33]. It is important to note that whilst levels of gentamicin were still found in the serum after 28 days, the levels of gentamicin did not reach systemic toxic levels in these animals [33]. The choice and concentration of the antibiotics chosen reflects previously reported clinical use in the literature. Important considerations should be made as to the choice of antibiotic to be mixed with any synthetic carrier, in order to protect the material from bacterial colonisation. Moreover, concentrations of the released antibiotic will be influenced by the content as well as by the size, surface, and composition of the carrier.

Comparative analysis of the Stimulan-G, Cerament-G and Herafill-G test materials revealed a similar increase in bone surface area and volume between animals implanted with Stimulan-G or Cerament-G test materials. Animals implanted with Herafill-G test materials demonstrated the lowest increases in bone volume and surface area of the test materials tested, at levels similar to the negative control animals in which the defect remained unfilled. These data are in line with previously published studies which have shown that calcium sulfate dissolves rapidly in vivo and stimulates new bone formation [34]. Samples size in the control arms of the study were small (n = 2) leading to large variances between replicates. This factor should be taken into consideration when interpreting these results. As such, the test materials require further investigation (with additional replicates) to establish their bone forming potential in addition to the mechanical strength of the filled defect.

Histologically, all materials appeared to be well tolerated over the duration of the experiment. However, differences were noted in the in vivo absorption of the three materials throughout the study. After 12 weeks, Stimulan-G defects were completely closed with mature bone and bone marrow. Only very small amounts of residual material remained. By contrast, whilst the Cerament-G material continued to absorb between 6 and 12 weeks, there was still residual material present after 12 weeks. Residual material was also present for defects filled with Herafill-G material which had a larger bead size, albeit at the centre of the defect which remained acellular. The data from this study was in keeping with the expected in vivo response to the implantation of calcium sulfate or its derivatives, which show that it is generally well tolerated [12]. Previous work has tried to elucidate the in-vivo absorption mechanism of these materials. Data from Ricci and colleagues demonstrated that calcium sulfate materials were absorbed by rapid dissolution both in vitro and in vivo, with absorption occurring from the outer surface inwards [35]. The absorption from the outside in was also observed in this study for the materials tested.

There are a number of limitations of the study, including the number of animals used. Furthermore, the performance of these materials in this animal model should not be taken as symptomatic of clinical efficacy in humans. The model used in this study is not designed to study infection, therefore the in-vivo efficacy of the antibiotic on protecting the materials from bacterial colonization could not be assessed, however other in-vitro studies have been successful in demonstrating this [36]. Future studies should include an assessment of other antibiotics as well as the concentration of these antibiotics, in combination with various bone graft materials on the in vivo response as well as comparing bone healing with each material with and without antibiotic to determine any effect of antibiotic on healing.
