# Author Correction: Comparative osteoconductivity of bone void fillers with antibiotics in a critical size bone defect model

**DOI:** 10.1007/s10856-021-06603-w

**Published:** 2021-10-06

**Authors:** Rema A. Oliver, Vedran Lovric, Chris Christou, William R. Walsh

**Affiliations:** Surgical and Orthopaedic Research Laboratories, UNSW Sydney, Prince of Wales Clinical School, Prince of Wales Hospital, Level 1 Clinical Sciences Building, Randwick, NSW Australia

Correction to: *Journal of Materials Science: Materials in Medicine* (2020) 31:80

10.1007/s10856-020-06418-1.

In Section 2.2 Surgery; the 3rd sentence contains a minor typographical error. The sentence should read as follows: ‘Following gaseous anaesthesia, 11 × 20 mm defects were created into the cancellous bone of the distal femur and proximal tibia.’

